# Age-related changes in susceptibility to false memories in different tasks

**DOI:** 10.3758/s13421-025-01778-x

**Published:** 2025-09-02

**Authors:** Aleea L. Devitt, Jeffrey Foster

**Affiliations:** 1 School of Psychological and Social Sciences, The University of Waikato, Private Bag 3105, Hamilton, 3240 New Zealand; 2 Department of Security Studies and Criminology, Macquarie University, Sydney, NSW Australia

**Keywords:** False memory, Memory distortion, Aging, DRM, Misinformation, Memory conjunction error

## Abstract

We become increasingly susceptible to various false memories as we age. Recent work has shown that in younger adults, associations between false memories in different paradigms are weak or non-existent. However, it is unknown whether the relationship between false memories changes for older adults. In two Experiments, we assessed false memories in younger and older adults elicited by three established false memory paradigms: the misinformation paradigm, the DRM (Deese-Roediger-McDermott) paradigm, and the memory conjunction tasks. We replicated previous findings of a lack of relationship between false memories arising from these tasks in younger adults, with the exception of raw false alarm rates in the DRM and memory conjunction tasks. In a novel finding, we extended this lack of relationship to older adults. In both Experiments, we observed a unique relationship between different types of false memories for older adults with higher compared with lower executive functioning capacity, which might reflect different recruitment of compensatory strategies. On the whole, our results concur with prior reports that different cognitive mechanisms underpin false memories in different paradigms, and care must be taken when generalizing results across memory paradigms, regardless of age.

**Supplementary Information:**

The online version contains supplementary material available at 10.3758/s13421-025-01778-x.

## Introduction

The constructive nature of memory means that we are susceptible to a range of different types of false memories. Understanding the prevalence, cause, and mechanisms of false memories is crucial; doing so provides insight into the cognitive underpinnings of the human memory system, and can help maximize memory accuracy. A variety of paradigms have been used to experimentally study false memories, including (but by no means limited to) the Deese-Roediger-McDermott (DRM) paradigm (Deese, 1959; Roediger & McDermott, 1995), the misinformation paradigm (Tousignant et al., 1986), the memory conjunction task (Lampinen et al., 2004; Odegard & Lampinen, 2004), and the imagination inflation task (Garry et al., 1996). However, recent work has shown that correlations between false memories elicited via these different paradigms are non-existent (Calvillo, 2014; Calvillo & Parong, 2016; Calvillo et al., 2019; Falzarano & Siedlecki, 2019; Monds et al., 2017; Ost et al., 2013; Otgaar & Candel, 2011; Patihis et al., 2018; Salthouse & Siedlecki, 2007; Wilkinson & Hyman, 1998) or at best weak (Ball et al., 2021; Bernstein et al., 2018; Lövdén, 2003; Murphy et al., 2023; Nichols & Loftus, 2019; Otgaar et al., 2012; Platt et al., 1998; Qin et al., 2008; Zhu et al., 2013). In other words, people who are particularly susceptible to one type of false memory are not necessarily more susceptible to other types of false memories. This lack of relationship suggests that different false memories are underpinned by different cognitive mechanisms (see, e.g., Hyman, 1999), and care must be taken when generalizing findings across different paradigms (see also Murphy et al., 2023, for a similar conclusion for episodic memory tasks more broadly).

To date, the relationship between different false memories has been examined primarily in children and younger adults. We become increasingly susceptible to false memories as we age, with older adults exhibiting higher rates of false memories compared to younger adults in most paradigms (see Devitt & Schacter, 2016, for review). However, to our knowledge, no study has examined more than one type of false memory within subjects in older adults. Maintaining an accurate memory with age is important for independent living and quality of life, an issue becoming increasingly important with an aging population (Ross & Schryer, 2015). Understanding how the relationship between different false memories changes across the lifespan could illuminate cognitive changes that lead to increased vulnerability to false memories as we get older. The current study is the first to draw links between different types of false memories in the same participants in an older adult sample. Across two experiments we examine the relationship between different types of false memories in younger and older adults.

### Aging and false memories in different paradigms

Older adults are more susceptible to false memories compared to younger adults in three common false memory paradigms. Age-related increases are seen in false memories in the DRM paradigm (e.g., Abichou et al., 2021; Balota et al., 1999; Koutstaal & Schacter, 1997; Norman & Schacter, 1997; Tun et al., 1998; but see Burnside et al., 2017; Pansuwan et al., 2020). Older adults are more susceptible to memory conjunction errors than younger adults across a range of stimuli, including words (Castel & Craik, 2003; Kroll et al., 1996; though see Jones & Jacoby, 2005; Matzen & Benjamin, 2013; Rubin et al., 1999), faces and names (Naveh-Benjamin et al., 2009), people and actions (Kersten & Earles, 2010; Kersten et al., 2008, 2013; Old & Naveh-Benjamin, 2008), and autobiographical memories (Devitt et al., 2016). Older adults are also more likely to fall prey to misinformation than younger adults (see Wylie et al., 2014).

The mechanisms driving false memories in each of these paradigms are, however, different, and there are several cognitive changes that might separately contribute to age-related increases in susceptibility to false memories in specific paradigms (see Devitt & Schacter, 2016, for a full discussion of these cognitive changes). For instance, false memories in the DRM paradigm are elicited by the semantic similarity of studied items. An overreliance on memory for general rather than specific stimulus features may account for the age-related increase in DRM errors, in that older adults are more susceptible to lures that are consistent with the semantic gist of the studied items (e.g., Koutstaal & Schacter, 1997). In contrast, the memory conjunction paradigm elicits false memories via the miscombination of elements of studied items. One reason for the age-related increase in conjunction error susceptibility is that memory for individual features of studied items is relatively preserved with age, whereas memory for the associations between those features declines (see Naveh-Benjamin, 2000; Naveh-Benjamin et al., 2003). Familiarity with the component pieces of conjunction stimuli in the absence of relational information may be misattributed as an indication that the conjunction stimulus has been previously encountered. Lastly, misinformation-based errors occur when inaccurate post-event information is considered to be part of the original event. Increased vulnerability to misinformation could be in part due to insufficient encoding or binding of contextual cues informing the source of the misleading information, resulting in misattribution of the misinformation.

It is possible that there is individual variability in the effects of aging on these different mechanisms contributing to false memories. For example, one person might experience an age-related decline in associative memory, leading to an increase in memory conjunction errors, whereas another might experience decline in memory for specific stimulus features, leading instead to more DRM errors. In this case, although on average there may be an increase in false memory susceptibility with age, for any one person increased susceptibility to one type of false memory is independent from other types. Like in younger adults, we would expect to see no relationship when correlating false memories across tasks for older adults.

Alternatively, source monitoring may be one stage of the encoding-retrieval process affected by cognitive aging that contributes more broadly to false memories (Johnson et al., 1993). With age comes a narrowing of effective monitoring strategies used. For example, we know that older adults can mitigate false memories when given retrieval support, but that they often do not engage these strategies spontaneously (Dodson et al., 2015; Henkel, 2008; Koutstaal, 2003; Thomas & Bulevich, 2006; Trelle et al., 2017). Above and beyond any binding deficits, older adults do not use recall-to-reject monitoring strategies – where information about originally encoded items is recalled to reject a lure item – as effectively as younger adults to reject conjunction lures (Fandakova et al., 2018). Older adults also experience difficulties with using multiple source cues to effectively monitor memory (Ferguson et al., 1992; Johnson et al., 1995), and with using different recollection-based monitoring processes (Gallo et al., 2006). Even when age-related impairments in recollection are mitigated, older adults still exhibit deficits in using recollected information to monitor memory (McDonough & Gallo, 2013). Age-related reductions in the efficiency of retrieval monitoring may result in a reliance on similar but less efficient monitoring strategies across tasks, such as relying on familiarity signals due to reduced recollection-based monitoring. Such a strategy could lead to increased false memories on all three paradigms mentioned above, and would result in a positive relationship between memory errors on those tasks.

In addition to aging, poor executive functioning ability – specifically working memory – has been linked more generally with increased susceptibility to different false memories, including in the DRM task (Peters et al., 2007; Unsworth & Brewer, 2010; Watson et al., 2005), misinformation task (Calvillo, 2014; Jaschinski & Wentura, 2002; Zhu et al., 2010), and memory conjunction task (Leding, 2012). In younger adults, those with high executive functioning ability are able to more effectively source monitor during memory retrieval (Ball et al., 2021; Unsworth & Brewer, 2010). Executive functioning ability declines with age, and could contribute to the age-related increase in susceptibility to various false memories (Butler et al., 2004; Glisky et al., 2001; Henkel et al., 1998; Plancher et al., 2009; Roediger & Geraci, 2007; Rubin et al., 1999). In particular, age-related declines in executive functioning have been linked with reductions in source monitoring (Thomas & McDaniel, 2013), and overreliance on familiarity when monitoring memory (Fandakova et al., 2013; Parkin & Walter, 1992). If age-related differences in monitoring strategies indeed contribute to a relationship between different types of false memories, it may not be aging per se that drives this relationship, but rather declining executive functioning. As such, in the current study we examine whether executive functioning ability predicts a distinct pattern of association between different types of false memories in younger and older adults.

### The current study

Across two experiments we examine whether the relationship between various false memories differs for younger and older adults. We assess false memories elicited by three different paradigms: the misinformation, the DRM, and the memory conjunction paradigms. Relationships between errors elicited in the misinformation and DRM paradigms have previously been examined in younger adults (Bernstein et al., 2018; Calvillo & Parong, 2016; Monds et al., 2017; Nichols & Loftus, 2019; Ost et al., 2013; Patihis et al., 2018; Zhu et al., 2013), but to our knowledge not in older adults. Moreover, the relationship between memory conjunction errors and false memories elicited by other paradigms has only been examined in one recent study in younger adults (Ball et al., 2021). As such, in Experiment 1, we examine the relationship between false memories in these three paradigms with an online sample of younger and older adults. In Experiment 2, we replicate these findings with an in-person sample of older adults.

For younger adults, we expected to replicate prior findings of weak to no relationship between false memories elicited via different paradigms. For older adults, we expected to see one of two outcomes. First, we expected a lack of relationship between false memories from different paradigms in older age if age-related changes in a range of cognitive mechanisms underpinning false memories contribute to individual differences in vulnerability to certain false memories. Alternatively, we expected a positive association between false memories from different paradigms if age-related changes in retrieval or monitoring strategies result in similar strategies being implemented across tasks. Finally, we predicted that executive functioning ability would distinguish two different trajectories of cognitive aging in relation to rates of false memories. If older adults with higher executive functioning use strategies in a similar way to that in younger adults, then we would expect them to show a similar relationship between false memories as younger adults (i.e., no association). In contrast, it is possible that older adults with poorer executive functioning use a more limited range of monitoring strategies on false memory tasks, resulting in a positive relationship in memory errors across tasks.

## Experiment 1

### Methods

#### Participants

We recruited 214 younger adults (aged 18–35 years) and 222 older adults (aged 65–92 years) online via Prolific, an online research participant pool. Participants completed two sessions, spaced approximately 1 day apart. Participants gave informed consent in a manner approved by the University of Waikato Human Research Ethics Committee, and were compensated with £3 per session completed. This study was preregistered on aspredicted.org (https://aspredicted.org/SDY_36J). Prior studies finding a significant relationship between false memories in the misinformation and DRM tasks in younger adults report an average correlation of 0.13 (0.12-0.14; Bernstein et al., 2018; Nichols & Loftus, 2019; Zhu et al., 2013). Following our first hypothesis that older adults will show a stronger positive relationship between false memory types than younger adults, a sample size of 150 per group will give us 80% power to detect a significant correlation of 0.20 or higher. Therefore, we aimed for a sample size of 150 participants per age group, and continued to collect data until we could meet this goal for all three false memory tasks.

Online participants were restricted geographically to the USA and UK. Of the younger adults, 93% specified that English was their first language, as did 97% of older adults. To verify age, we asked a speeded question in each session, where participants had 6 s to type in their year of birth. In total we excluded 48 younger adults and 34 older adults. As preregistered, we excluded participants who either: (1) failed two or more attention checks (1 younger and 1 older adult); (2) answered “no” to the question “should we use your data?” (5 younger and 2 older adults); (3) provided a year of birth inconsistent with their specified age (3 younger and 2 older adults); (4) achieved at or below chance accuracy on a memory task (based on proportion correct for studied and new items; 38 younger and 10 older adults); or (5) indicated that they had a history of dementia or stroke (1 younger and 9 older adults). We also excluded ten older adults who took the study twice. Several participants did not return for session 2, or had technical difficulties with the executive functioning task. Of those who completed session 2, an additional five younger and 11 older adults’ data were excluded from session 2 exclusively, either because they did not complete the DRM task due to technical difficulties, or because they achieved chance performance on the DRM task. As such, 166 younger adults and 188 older adults were included in the misinformation and memory conjunction analyses, 151 younger adults and 151 older adults were included in the DRM analyses, and 147 younger adults and 144 older adults were included in the executive functioning analyses. The mean age for younger adults was 26 years (*SD* = 5.03), and for older adults was 70 years (*SD* = 4.98). Younger and older adults had similar years of education (younger *M* = 15.66 years, *SD* = 3.25; older *M* = 15.13, *SD* = 3.32; *t*(344) = 1.49, *p* =.137).

#### Stimuli

All stimuli were presented via Qualtrics.

*Misinformation task:* The misinformation task comprised three stages. (1) *Encoding*, in which participants viewed a silent video lasting 6 min 28 s of an electrician stealing personal property from a client’s home (Takarangi et al., 2006). There were two versions of the video where critical items differed (e.g., the electrician drank Coke in one video and Pepsi in the other), which were counterbalanced across participants. (2) *Narrative*, in which participants read a narrative account about the video which contained misinformation. Narratives were read one sentence at a time and were self-paced. The narrative contained four misinformation items, where information in the narrative conflicted with information in the video (e.g., if the electrician drank Coke in the video, the narrative stated that he drank Pepsi). The narrative also contained four control items, where the narrative information did not conflict with the video (e.g., the narrative stated that the electrician drank a soda). There were two narrative versions for each video, in which the misinformation and control items were reversed, which were counterbalanced across participants. (3) *Recognition*, in which participants completed a two-alternative forced-choice recognition task to identify which of the two items were presented in the original video. There were 20 questions: 12 filler questions where the same information was provided in the video and the narrative, four critical questions where misinformation was provided in the narrative, and four control questions where the information from the narrative did not conflict with the video.

*Memory conjunction task:* During the *encoding* stage 96 conjunction words were presented (e.g., SNOWMAN, SANDCASTLE; adapted from Leding et al., 2007). Participants were instructed to remember these words for a later memory test, then each word was shown one at a time for 2 s. Words were presented in three lists of 32 words each, so that parent conjunctions were presented relatively close together in time. The *recognition* stage involved 96 conjunction words: 24 intact (studied) words, 24 conjunction lure words (in which both parts of the conjunction were taken from studied items, e.g., SANDMAN), 24 feature lures (in which one part of the conjunction was taken from a studied item, the other from a new word, e.g., SNOWBALL), and 24 entirely new words. Participants determined whether each word had been studied previously (“old”) or not (“new”). The recognition test was self-paced.

*DRM:* During the *encoding* stage eight lists of 12 words each were presented (96 words total; adapted from Stadler et al., 1999). Participants were instructed to remember these words for a later memory test, then words were shown one at a time for 2 s each. Each list contained words that were semantically related to a non-presented critical lure (critical lures used were SLOW, CAR, SLEEP, SWEET, MOUNTAIN, NEEDLE, ANGER, SMELL). The *recognition* stage involved 32 words: eight studied words (the first word of each list), eight critical lure words, and 16 new words (eight words from alternative non-presented lists, and eight critical lure words from those non-presented lists). Participants determined whether each word had been studied previously (“old”) or not (“new”). The recognition test was self-paced.

*Running span:* Participants completed two running span tasks as a measure of working memory capacity (adapted from Broadway & Engle, 2010). In an icon span task, participants saw symbols appearing one at a time for 1 s each, and were asked to identify the last *n* items in the exact order they were presented in, out of a pool of 12 possible items (*n* = 2–6, random order). Similarly, in a location span task, participants saw several squares in a 4 × 4 grid. Several squares were highlighted one at a time for 1 s each, after which participants were asked to report the location of the last *n* flashing squares by clicking on the grid (*n* = 2–6, random order). In both tasks, one point was given for each correctly chosen item or location, in the correct order.

### Procedure

**Fig. 1:**
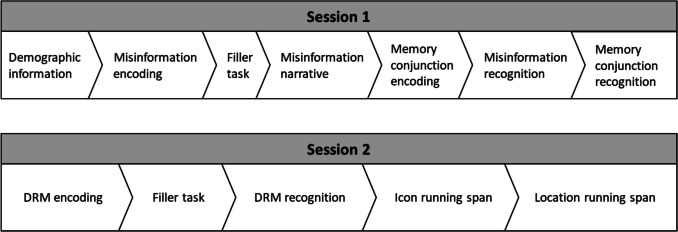
Order of tasks across the two experimental sessions of Experiment 1

All participants completed the tasks in the same order, to minimize time spent on the task and participant dropout (see Fig. 1). Each session took approximately 25 min to complete.

*Session 1:* (1) Demographic information was collected. (2) Misinformation encoding stage. (3) A filler task involving matching pairs of symbols on virtual cards (2 min); (4) Misinformation narrative stage. (5) Memory conjunction encoding stage. (6) Misinformation recognition stage. (7) Memory conjunction recognition stage. (8) Speeded year of birth question.

*Session 2: *(1) DRM encoding stage. (2) A filler task involving identifying instances of the letter “B” from a letter array (1 min). (3) DRM recognition stage. (4) Icon running span. (5) Location running span. (6) Speeded year of birth question.

#### Analysis

For the misinformation task, false memories were calculated as the proportion of misled items that were identified as being presented in the video. For the memory conjunction and DRM tasks, false memories were calculated as the proportion of conjunction lure words or critical lure words that were recognized as “old.” We also used corrected false memory rates for the memory conjunction and DRM tasks, which were calculated as proportion of false alarms to conjunction or critical lures minus proportion of false alarms to new items (see Leding, 2012; McCabe & Smith, 2002). Executive functioning scores were calculated by summing the number of items that were correctly recalled in the original order during the running span tasks. A composite measure of executive functioning was calculated by z-scoring and averaging these scores across the two running span tasks (Foster et al., 2015).

We ran an analysis of variance (ANOVA) in each false memory task to compare younger and older adult groups on the number of hits and false memories produced. Where sphericity was violated, we used the Greenhouse-Geisser correction. To compare younger and older adult groups on overall number of corrected false memories produced in each task, we ran a multivariate ANOVA (MANOVA). To examine the relationship between the proportion of hits and false memories made in each task, we conducted Pearson’s correlations, separately for younger and older adults. We applied a Bonferroni correction for multiple comparisons to each group of correlations; correlations are only reported as significant if they survived the corrected alpha level. To assess whether the relationship between false memories differed according to executive functioning ability, we ran a multiple regression for each pairing of false memories, with the executive functioning composite score specified as a continuous measure, age group as a categorical measure, and all interactions specified.^1^

### Results

#### Overall false memories

*Reliability: *The three tasks reliably produced false memories for both younger and older adults. For the misinformation task, only six younger and seven older adults did not endorse any misled items. For the memory conjunction task, one older adult did not false alarm to any conjunction words. For the DRM task, two younger and two older adults did not false alarm to any critical lures.

Reliability was not computed for misinformation errors because of a low number of misinformation items which were counterbalanced across participants. To assess the internal reliability of the memory conjunction and DRM tasks, we examined Cronbach’s α. Reliability for conjunction lures in the memory conjunction task was α = 0.75 for younger adults and α = 0.74 for older adults. Reliability for critical lures in the DRM paradigm was α = 0.66 for younger adults and α = 0.65 for older adults. The reliability for the DRM task is comparable to Nichols and Loftus (2019) and Ost et al. (2013), and across the two tasks indicates that reliability was sufficient.

#### Age differences in false alarms

*Misinformation:* See Table 1 for mean proportion of responses in each memory task. A 2 × 2 repeated-measures ANOVA with item type (misled, control) and age group (younger adults, older adults) revealed a main effect of item type (F(1,352) = 275.91, *p* <.001). Participants made more false alarms for misled compared to control items, consistent with a misinformation effect. There was no main effect of age group (F(1,352) = 0.03, *p* =.863) or age-by-item type interaction (F(1,352) = 2.44, *p* =.12).

**Table 1:** Mean proportion of responses in each memory task by age group (standard deviations in parentheses)

Task	Response type	Item type	Younger adults	Older adults Experiment 1	Older adults Experiment 2
Misinformation	Hit	Congruous item	0.90 (0.10)	0.93 (0.08)	0.94 (0.07)
	False alarm	Misled item	0.58 (0.27)	0.61 (0.24)	0.51 (0.26)
		Control item	0.32 (0.24)	0.29 (0.22)	0.24 (0.20)
Memory conjunction	Hit	Studied item	0.67 (0.16)	0.70 (0.16)	0.63 (0.18)
	False alarm	Conjunction lure	0.47 (0.18)	0.45 (0.18)	0.45 (0.21)
		Feature lure	0.38 (0.19)	0.33 (0.17)	0.33 (0.20)
		New lure	0.32 (0.19)	0.26 (0.18)	0.30 (0.21)
		Corrected conjunction	0.15 (0.14)	0.19 (0.13)	0.14 (0.18)
DRM	Hit	Studied item	0.82 (0.19)	0.87 (0.15)	0.83 (0.13)
	False alarm	Critical lure	0.67 (0.25)	0.71 (0.23)	0.65 (0.19)
		New	0.19 (0.18)	0.08 (0.12)	0.22 (0.15)
		Corrected critical	0.48 (0.29)	0.63 (0.24)	0.43 (0.25)

*Memory conjunction:* A 3 × 2 repeated-measures ANOVA with lure type (conjunction, feature, new) and age group (younger adults, older adults) revealed a main effect of lure type (F(1.95,686.87) = 287.55, *p* <.001). More false alarms were made for conjunction versus feature and new lures, and for feature versus new lures (ps <.001), demonstrating that the conjunction task resulted in memory conjunction errors. There was a main effect of age group (F(1,352) = 5.97, *p* =.015), with younger adults making more false alarms overall compared to older adults. However, a significant age-by-lure type interaction was also seen (F(1.95,686.87) = 3.88, *p* =.022), whereby younger adults made more false alarms specifically to new lures compared to older adults (*p* =.049).

*DRM:* A 2 × 2 repeated-measures ANOVA with lure type (critical, new) and age group (younger adults, older adults) revealed a main effect of lure type (F(1,300) = 1329.3, *p* <.001). Participants made more false alarms for critical lures versus new lures, demonstrating that the DRM task resulted in false memories. There was no main effect of age (F(1,300) = 3.22, *p* =.074). However, a significant age-by-lure type interaction was seen (F(1,300) = 21.6, p <.001). Younger and older adults made a similar proportion of false alarms to critical lures, but younger adults made more false alarms to new lures compared to older adults (*p* <.001).

A MANOVA was conducted to examine age differences in false alarm rates across the three tasks. Because younger adults tended to make more false alarms to new lures, we used the corrected rates of false alarms for the memory conjunction and DRM tasks. Older adults were more susceptible to false alarms overall compared to younger adults (Pillai’s Trace =.08, *F*(3,298) = 9.06, *p* <.001). Pairwise comparisons revealed no age differences in endorsement of misled items on the misinformation task (*p* =.177), but older adults made more false alarms than younger adults in the memory conjunction (*p* =.038) and DRM tasks (*p* <.001).

#### Age differences in hit rates

To assess age differences in hit rates across the three false memory tasks, we ran a series of independent-samples t-tests. Compared with younger adults, older adults had a higher rate of hits on the misinformation (*t*(352) = 2.49, *p* =.01) and DRM tasks (*t*(300) = 2.59, *p* =.01; see Table 1 for means). No age differences were found on the memory conjunction task (*t*(352) = 1.45, *p* =.15).

#### Relationship between false memories

We first replicated prior findings looking at the relationship between different false memories in younger adults by running Pearson’s correlations between the proportion of false memories elicited in the three memory tasks (see Table 2). Consistent with prior research, there was no significant relationship between false memories in the misinformation task and either the memory conjunction task or the DRM task. However, a positive relationship was observed between false memories in the memory conjunction and DRM tasks. A significant positive relationship was also found between false alarms to new lures in memory conjunction and DRM tasks (*r* = 0.47, *p* <.001), suggesting that the relationship between DRM and memory conjunction tasks may be driven by similarities in response bias – the level of memory evidence required to make an “old” response – rather than susceptibility to false memories per se, because both tasks involved an old/new recognition judgement (unlike the misinformation task). To account for this possibility, we ran the above correlations using corrected false memory rates for the memory conjunction and DRM tasks, and found no relationships between false memories in any of the tasks.

**Table 2:** Pearson’s correlations between the proportion of false memories and true memories (hit rates) elicited in the three memory tasks in Experiment 1

	Misinformation	Memory conjunction	Corrected memory conjunction
*False memories*			
**Younger adults**			
Memory conjunction	0.11		
DRM	−0.01	**0.39**	
Corrected memory conjunction	−0.04		
Corrected DRM	−0.09		0.06
**Older adults**			
Memory conjunction	0.03		
DRM	−0.03	0.19	
Corrected memory conjunction	0.07		
Corrected DRM	−0.08		0.10
*True memories*			
**Younger adults**			
Memory conjunction	0.01		
DRM	0.13	**0.46**	
Corrected memory conjunction	0.09		
Corrected DRM	0.10		**0.37**
**Older adults**			
Memory conjunction	0.10		
DRM	−0.04	0.17	
Corrected memory conjunction	0.15		
Corrected DRM	−0.02		**0.27**

Note. Correlations in bold are statistically significant at *p* = .008, surviving a Bonferroni correction for multiple comparisons

Next, we ran Pearson’s correlations between the proportion of false memories elicited in the three false memory tasks in the older adult group (see Table 2). Similar to younger adults, there was no significant relationship between false memories in the misinformation task and either the memory conjunction task or the DRM task. No relationship was found between false memories in the memory conjunction and DRM tasks using either corrected or uncorrected rates. Like younger adults, older adults demonstrated a significant positive relationship between false alarms to new lures in the memory conjunction and DRM tasks (*r* = 0.37, *p* <.001).

#### Relationship between true memories

We also ran correlations between true memories (hit rates) on each of the three tasks (see Table 2). Because for the misinformation task there are more true trials than misled trials, we randomly selected four congruous trials so as to be able to fairly compare true memory relationships with the false memory relationships. There was a positive relationship between uncorrected hit rates on the MCE and DRM tasks for younger adults, and between the corrected hit rates on these tasks for both younger and older adults (all *p*s <.001).

#### Executive functioning ability

As expected, older adults had lower composite executive functioning scores (*M* = −0.47, *SD* = 0.82) compared to younger adults (*M* = 0.40, *SD* = 0.74; *t*(289) = 9.58, *p* <.001). We used Pearson’s correlations to examine the relationship between false memories and executive functioning separately for younger and older adults. No relationships were found between false memories in any of the three tasks and executive functioning score for either age group (*r*s <.16, *p*s >.057).

To examine whether the relationship between false memories differed according to executive functioning ability, we ran a regression for each pairing of false memories (using corrected proportion for DRM and memory conjunction errors), with the executive functioning composite score specified as a continuous measure, and age group as a categorical measure. In particular, we were interested in whether there was an interaction between executive functioning score, false memories, and age group. The model of misinformation errors predicting memory conjunction errors was not significant (*R*^2^ = 0.02, *F*(7, 283) = 1.02, *p* =.416). The model of misinformation errors predicting DRM errors was significant (*R*^2^ = 0.09, *F*(7, 283) = 3.76, *p* <.001), but this was driven by a significant effect of age group, where older adults produced more DRM errors than younger adults (*b* = −0.15, *SE* = 0.04, 95% CI = [−0.22, −0.08], *t*(283) = −4.20, *p* <.001).

The model with memory conjunction errors predicting DRM errors was significant (*R*^2^ = 0.11, *F*(7, 283) = 4.92, *p* <.001). Of interest for this analysis, there was a significant three-way interaction between executive functioning, age group, and memory conjunction rate in predicting DRM errors (see Table 3). To investigate this interaction, we split the sample into younger and older adults, and ran simple-effects tests separately in each age group. For younger adults, there was no relationship between memory conjunction errors and DRM errors regardless of executive functioning score. However, for older adults, the relationship between memory conjunction errors and DRM errors was moderated by executive functioning score (see Table 4). Older adults with higher executive functioning scores exhibited a positive relationship between the two error types, whereas older adults with lower executive functioning scores did not.

**Table 3:** Multiple regression model of predictors for errors in the DRM paradigm

Predictor	*b* [95% CI]	*SE*	*t*	*p*
(Intercept)	0.51 [0.46, 0.56]	0.03	18.78	<.001
Memory conjunction errors	0.28 [0.03, 0.53]	0.13	2.21	.028
Age group	−0.11 [−0.22, −0.01]	0.05	2.09	.038
Executive functioning score	−0.02 [−0.08, 0.04]	0.03	0.65	.514
Age group × Memory conjunction errors	−0.27 [−0.76, 0.23]	0.25	1.05	.294
Age group × Executive functioning score	0.17 [0.05, 0.29]	0.06	2.75	.006
Memory conjunction errors × Executive functioning score	0.22 [−0.08, 0.53]	0.15	1.46	.146
Age group × Memory conjunction errors × Executive functioning score	−0.75 [−1.35, −0.15]	0.31	2.45	.015

**Table 4:** Simple effects of memory conjunction errors and executive functioning score predicting DRM errors, separated by age group

Age group	Executive functioning score	*b*	*SE*	*t*	***p***
Younger adults	1 *SD* below the mean	0.29 [−0.39, 0.96]	0.34	0.83	.405
	Mean	0.15 [−0.21, 0.51]	0.18	0.83	.409
	1 *SD* above the mean	0.02 [−0.39, 0.42]	0.21	0.08	.937
Older adults	1 *SD* below the mean	−0.14 [−0.52, 0.24]	0.19	0.72	.473
	Mean	0.40 [0.05, 0.74]	0.18	2.24	.026
	1 *SD* above the mean	0.93 [0.37, 1.49]	0.29	3.24	.001

### Discussion

In Experiment 1 we found no relationship between false memories in the misinformation and DRM tasks for younger adults, replicating previous findings and extending them to an online sample. We did find a positive relationship between false alarms on the DRM and memory conjunction tasks for younger adults. Both paradigms required an old/new recognition response, whereas the misinformation task used a forced-choice response. As such, the relationship between the memory conjunction and DRM tasks could be accounted for by a similar bias towards responding “old” on the two tasks. Previous research suggests that such a response bias is a stable individual difference that correlates on tasks with different stimuli (Kantner & Lindsay, 2012). Our finding that false alarms to new lures correlates across the memory conjunction and DRM tasks is consistent with this view. When we use corrected rates of false alarms to account for this bias, the two tasks are no longer related. Note that all three memory tasks produced rates of false memories as expected based on prior studies, validating the online format. The DRM and memory conjunction tasks had acceptable internal reliability for false memories, reducing the likelihood that null correlations are false negatives (i.e., Type II errors; Ost et al., 2013).

We also examined the relationship between true memories (hit rates) across the three tasks. An association was found between the memory conjunction and DRM tasks; however, true memories in the misinformation task were not associated with the other two. Note that the congruent items in the misinformation task serve as distractors, not as an indication of accurate memory per se. As a result, there was little variation in hit rates in the misinformation task (90% of participants in Experiment 1 scored 10+ out of 12 congruous items), which might contribute to these null findings. However, when using the control items as an indicator of accurate memory, still no significant associations emerged. We discuss these findings further in the *General discussion*.

The main aim of the current study was to examine the relationship between false memories in various tasks in older adults, to see whether this differed from the lack of relationship found in younger adults. Critically, older adults also demonstrated no relationship between false memories in the three memory tasks, while still showing an association between true memories in the memory conjunction and DRM tasks. Our secondary aim was to examine the contribution of executive functioning ability to false memories in different paradigms. In contrast to prior findings (Calvillo, 2014; Jaschinski & Wentura, 2002; Leding, 2012; Peters et al., 2007; Unsworth & Brewer, 2010; Watson et al., 2005; Zhu et al., 2010), we observed no relationship between executive functioning ability and false memories in any of the three tasks. While we did find a distinct pattern of association between different false memories depending on executive functioning ability, this was in the opposite direction to that expected. Older adults with low executive functioning ability displayed no relationship between different false memories, whereas older adults with high executive functioning scores exhibited a positive relationship between false memories in the memory conjunction and DRM tasks. Given this finding was unexpected, we aimed to replicate these results in Experiment 2 with additional executive functioning tests.

Notably, in Experiment 1 we did not find the expected age-related increase in raw rates of false memories in any of the three memory tasks. Instead, younger adults were more susceptible than older adults to new lures in both the memory conjunction and DRM tasks, suggesting that younger adults were more liberal with their old responses. This pattern of results is not unusual for an online sample, where younger adults may be less motivated or attentive, and older adults are likely to be particularly high performing and experienced with memory tests (Greene & Naveh-Benjamin, 2022; Ogletree & Katz, 2021). As such, in Experiment 2 we also assessed whether the lack of relationship between false memories for older adults holds in an in-person format. Additionally, in Experiment 2 we included a measure of everyday cognitive failures, to examine the potential real-world effect of different memory distortions. This analysis was largely exploratory, and we did not have strong hypotheses regarding whether one type of memory error might be associated with everyday cognitive failures more than another. Finally, it is possible that the static order and interleaved nature of the tasks in Experiment 1 could have led to interference between these tasks (although the lack of correlation between false memories does not support this possibility). In Experiment 2 the order of the three false memory tasks was counterbalanced across participants, to ensure that order effects or overlapping tasks were not unduly influencing the results.

## Experiment 2

### Methods

#### Participants

We recruited 49 older adults (aged 65–85 years) via postings around the Hamilton area in Waikato, New Zealand. Participants came into the lab to complete a single experimental session. Participants gave informed consent in a manner approved by the University of Waikato Human Research Ethics Committee, and were compensated with $40. We excluded three participants for reporting a history of neurological conditions. Given one of our aims was to replicate the relationship between memory conjunction and DRM errors for those with high executive functioning as found in Experiment 1, a sample size of 37 gives us 80% power to detect a significant correlation of 0.40 or higher. While we aimed to collect 37 participants per executive functioning group (high and low), data collection was halted by the COVID-19 restrictions. As such, 45 older adults were in the final sample, with a mean age of 70.69 years (*SD* = 4.66). Of these participants, 98% spoke English as a first language. Note that due to the need to collect data before COVID-19 restrictions, Experiment 2 was not preregistered.

#### Stimuli

As in Experiment 1, participants completed the misinformation, memory conjunction, DRM, and running span tasks. The misinformation, memory conjunction, and running span tasks remained the same as in Experiment 1. However, we presented more words in the DRM task to increase potential false alarms to critical lures. During the *encoding* stage 12 lists of 12 words each were presented (144 words total, an increase from 96 in Experiment 1). Additional critical lures used were SMELL, RIVER, COLD, ROUGH, RUBBER. The *recognition* stage involved 48 words: 12 studied words (the first word of each list), 12 critical lure words, and 24 new words (12 words from alternative non-presented lists, and 12 critical lure words from those non-presented lists). All other aspects of the DRM task were the same as in Experiment 1.

Participants also completed several additional tasks and questionnaires. Participants completed the mini-Addenbrooke’s Cognitive Examination (Mini-ACE); a 5-min questionnaire that screens for possible mild cognitive impairment or dementia.

We included three subtests from the Delis-Kaplan Executive Function System test battery (D-KEFS; Delis et al., 2001) that load onto a monitoring, inhibition, and conceptual flexibility factor, respectively (Latzman & Markon, 2010; Li et al., 2015). (A) Verbal fluency category: participants generate as many words as possible in accordance with set rules, switching between fruit and furniture. Scores used were number of correct words (total fluency) and accuracy (total switching). (B) Color-word interference: participants are presented with colored words, and must name the color of the ink each word is written in, rather than the word itself. The measures used were the time taken to name words in the inhibition and switching conditions. (C) Card sorting free sort: participants are given cards with different shapes and words on them, which must be sorted into different groups according to set rules. Scores used were the number of correct total sorts, and the total description score.

We assessed the subjective experience of everyday cognitive functioning and slips using the Cognitive Failures Questionnaire (Broadbent et al., 1982). Participants responded to each of 25 items (e.g., do you find you forget appointments?) with their experience over the last 6 months, using a 5-point scale (0 = never, 4 = very often). Responses on all items are summed. Overall scores vary between 0 and 100, with higher scores representing more cognitive failures.

All stimuli were presented via Qualtrics, except the Mini-ACE and the D-KEFS, which were completed with paper and pencil. The session took around 2 h to complete.

#### Procedure

**Fig. 2:**
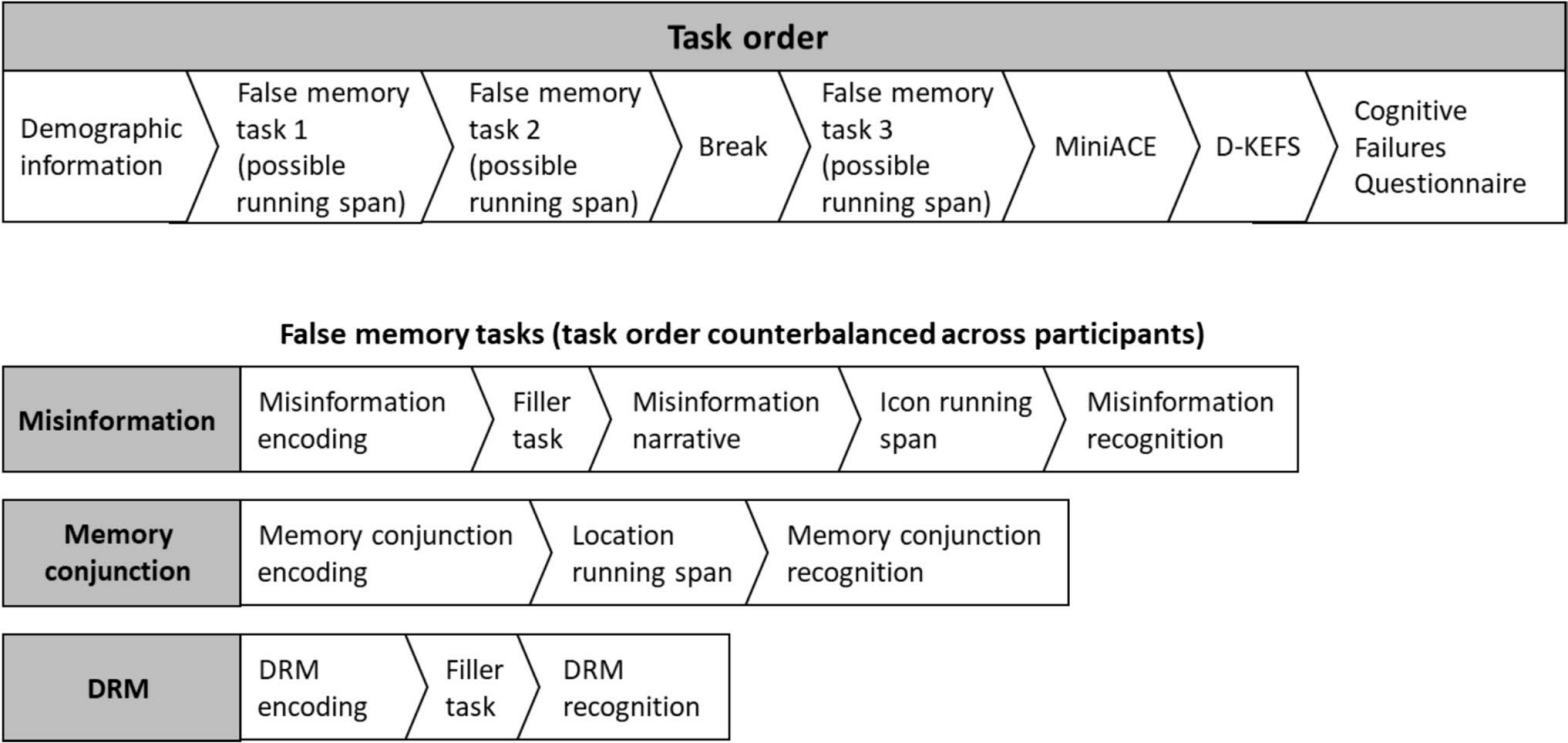
Order of tasks in Experiment 2

In a departure from the fixed order of tasks in Experiment 1, in Experiment 2 the order of the three false memory tasks was counterbalanced across participants. The location and icon running span tasks were conducted between encoding and recognition for the misinformation and memory conjunction tasks. As such, the procedure for the session was as follows: (1) Demographic information was collected. (2) False memory task 1 (and possible running span). (3) False memory task 2 (and possible running span). (4) A 10-min break if necessary. (5) False memory task 3 (and possible running span). (6) Mini-ACE. (7) D-KEFS. 8) Cognitive Failures Questionnaire (see Fig. 2 for more detail).

#### Analysis

False memories and executive functioning scores based on the running span tasks were calculated in an identical manner to Experiment 1. For DKEFS measures, the raw scores within each task were converted to z-scores, then averaged to form separate composite scores for each factor (monitoring, inhibition, and conceptual flexibility).

As in Experiment 1, we conducted Pearson’s correlations to examine the relationship between the proportion of hits and false memories made in each task. We applied a Bonferroni correction for multiple comparisons to each group of correlations, and correlations are only reported as significant if they survived the corrected alpha level. To assess whether the relationship between false memories was different according to executive functioning ability, we ran a multiple regression for each pairing of false memories, together with the executive functioning composite score (determined via running span) specified as a continuous measure, and a false memory × executive functioning interaction term. We repeated this analysis for each of the three DKEFS composite scores.

### Results

#### Overall false memories

*Reliability:* Again the three tasks reliably produced false memories. For the misinformation task, only three older adults did not endorse any misled items. All older adults made at least one false alarm to conjunction lures and critical lures in the memory conjunction and DRM tasks, respectively.

As in Experiment 1, reliability was not computed for misinformation errors because of a low number of misinformation items which were counterbalanced across participants. We examined Cronbach’s α to assess the internal reliability in the memory conjunction and DRM tasks. Reliability was good for conjunction lures in the memory conjunction task (α = 0.81). However, reliability for critical lures in the DRM task was low compared to previous studies (α = 0.59; Nichols & Loftus, 2019; Ost et al., 2013). We continued with the analyses below, but caution is warranted in interpreting these results (see *General discussion* for more).

#### False alarm rates

All memory tasks elicited false alarm rates as expected (see Table 1 for mean proportion of responses in each memory task). In the misinformation task, participants made more false alarms for misled compared with control items, consistent with a misinformation effect (*t*(44) = 5.42, *p* <.001). In the memory conjunction task, a repeated-measures ANOVA revealed a main effect of lure type (*F*(2,88) = 19.5, *p* <.001). Pairwise comparisons show that participants made more false alarms to conjunction lures compared to feature lures (*p* <.001) and new lures (*p* <.001). However, there was no difference in false alarms to feature and new lures (*p* = 0.48). Finally, in the DRM task, participants made more false alarms to critical lures than new lures (*t*(44) = 11.70, *p* <.001).

#### Relationship between false memories

We aimed to replicate the older adult findings from Experiment 1 showing no significant relationship between false memories elicited by different tasks. To do this, we ran Pearson’s correlations between the proportion of false memories elicited in each of the three memory tasks (see Table 5), using both corrected and uncorrected rates of false memories for the memory conjunction and DRM tasks. There were no significant relationships between false memories in any of the three tasks. A positive correlation was found between false alarms to new lures in the memory conjunction and DRM tasks (*r* = 0.31, *p* =.03).

**Table 5:** Pearson’s correlations between the proportion of false memories and true memories (hit rates) elicited in the three memory tasks for older adults in Experiment 2

	Misinformation	Memory conjunction	Corrected memory conjunction
***False memories***			
Memory conjunction	−0.04		
DRM	−0.13	0.04	
Corrected memory conjunction	−0.33		
Corrected DRM	−0.05		−0.06
***True memories***			
Memory conjunction	−0.27		
DRM	0.10	0.16	
Corrected memory conjunction	−0.15		
Corrected DRM	0.33		0.08

#### Relationship between true memories

We also ran correlations between true memories (hit rates) on each of the three tasks (see Table 5). As with Experiment 1, we randomly selected four congruous trials in the misinformation task so as to be able to fairly compare true memory relationships with the false memory relationships. We found no associations between the three tasks, for either corrected or uncorrected rates of memory conjunction and DRM errors.

#### Executive functioning ability

First, we used Pearson’s correlations to examine the relationships between the executive functioning scores (the running span task, and three DKEFS subtypes: monitoring, inhibition, and conceptual flexibility), to determine whether they assessed different constructs. Note that due to technical errors, only 39 participants completed the running span tasks. Monitoring was positively correlated with inhibition (*r* =.52, *p* <.001), conceptual flexibility (*r* =.46, *p* =.001), and running span (*r* =.46, *p* =.003). Correlations between the other executive functioning scores did not survive a Bonferroni correction of α <.013 (*r*s <.35, *p*s >.019).

We next used Pearson’s correlations to examine the relationship between false memories and executive functioning scores. As with Experiment 1, no relationships were found between false memories in any of the three false memory tasks and running span scores, or the DKEFS subtypes (see Table 6).

**Table 6:** Pearson’s correlations between the proportion of false memories elicited in the three memory tasks and executive functioning scores in Experiment 2

	Misinformation	Memory conjunction (corrected)	DRM (corrected)
Running span	−0.07	0.04	−0.01
Monitoring	−0.24	0.10	0.22
Inhibition	−0.05	0.08	0.16
Conceptual flexibility	−0.16	−0.08	−0.004

Replicating the analysis from Experiment 1, to examine whether the relationship between false memories differed according to executive functioning ability, we ran a regression for each pairing of false memories (using corrected proportion for DRM and memory conjunction errors), with the executive functioning (running span) composite score included as a predictor, and a false memory × executive functioning interaction term. The model of misinformation errors predicting DRM errors was not significant (*R*^2^ = 0.02, *F*(3, 35) = 0.27, *p* =.847). Unlike Experiment 1, the model with memory conjunction errors predicting DRM errors was not significant (*R*^2^ = 0.11, *F*(3, 35) = 1.48, *p* =.24). Also unlike Experiment 1, the model of misinformation errors predicting memory conjunction errors was significant (*R*^2^ = 0.29, *F*(3, 35) = 4.85, *p* =.006). Specifically, there was a significant interaction between misinformation errors and executive functioning score (see Table 7). Simple-effects tests reveal that for older adults with low executive functioning score, there was no relationship between misinformation and memory conjunction errors. But as executive functioning score increases, a negative relationship emerges between these two errors (see Table 8). We repeated these analyses using the three DKEFS composite scores (monitoring, inhibition, and conceptual flexibility) in place of the running span score, and the results were replicated for the monitoring score (see Online Supplementary Material).

**Table 7:** Multiple regression model of predictors for errors in the memory conjunction paradigm

Predictor	*b* [95% CI]	*SE*	*t*	*p*
(Intercept)	0.13 [0.08, 0.18]	0.03	4.86	<.001
Misinformation errors	−0.24 [−0.45, −0.03]	0.10	2.29	.028
Executive functioning score	−0.004 [−0.06, 0.05]	0.03	0.15	.881
Misinformation errors × Executive functioning score	−0.35 [−0.61, −0.09]	0.13	2.72	.01

**Table 8:** Simple effects of misinformation errors and executive functioning score predicting memory conjunction errors

Executive functioning score	*b*	*SE*	*t*	*p*
1 *SD* below the mean	0.10 [−0.25, 0.45]	0.17	0.58	.564
Mean	−0.23 [−0.44, −0.02]	0.10	2.15	.033
1 *SD* above the mean	−0.56 [−0.86, −0.26]	0.15	3.8	<.001

#### Relationship with Cognitive Failures Questionnaire (CFQ)

No relationships were found between CFQ scores and rates of false memories in any of the three tasks, for either uncorrected or corrected false alarm rates (*r*s < −0.32, *p*s >.03; note that correction for multiple comparisons α =.01).

### Discussion

In Experiment 2 we attempted to replicate the lack of relationship between false memories elicited via different tasks using a sample of older adults run in an in-person format. As in Experiment 1, no relationship was found between false memories elicited via different tasks. Yet unlike Experiment 1, no associations were found between true memories on any of the tasks. Moreover, false memories were not associated with a measure of everyday cognitive failures, the CFQ. However, given the DRM task did not have acceptable internal reliability for false memories, caution is advised in interpreting null correlations involving the DRM task.

We also aimed to replicate the differential relationship between memory conjunction and DRM errors for older adults with high and low executive functioning ability; however, this result was not replicated. Instead, we found a negative relationship between false memories in the misinformation task and the memory conjunction task for older adults with high executive functioning ability (as determined by running span and monitoring ability).

## General discussion

In two experiments, we examined whether the relationship (or lack thereof) of false memories arising from different paradigms changes with age, given general age-related increases in susceptibility to false memories. Replicating previous findings, for younger adults we found no relationship between false memories in the misinformation, DRM, and memory conjunction tasks, with the exception of raw false alarm rates in the DRM and memory conjunction tasks. In a novel finding, older adults also demonstrated no relationship between false memories in the three memory tasks. These results provide further evidence that for the most part, different cognitive mechanisms underpin false memories in different paradigms (Bernstein et al., 2018; Calvillo, 2014; Calvillo & Parong, 2016; Calvillo et al., 2019; Monds et al., 2017; Nichols & Loftus, 2019; Ost et al., 2013; Patihis et al., 2018; Qin et al., 2008; Wilkinson & Hyman, 1998; Zhu et al., 2013).

### Replicating no relationship between false memories in younger adults

Our first aim was to replicate prior findings demonstrating weak to no relationships between false memories elicited via different tasks using an online sample of younger adults. However, a positive relationship was apparent between false alarms on the DRM and memory conjunction tasks, which is consistent with prior work looking at these two tasks (Ball et al., 2021). It is possible that this relationship reflects the shared internal generation of errors on the DRM and memory conjunction tasks, in contrast to the misinformation task where errors are externally suggested. Alternatively, because both tasks required an old/new recognition response (again in contrast to the misinformation task), this relationship might reflect individual differences in response bias, in that people who tend to respond “old” in one task also respond “old” in another task. In other words, people with a more liberal response bias might consider a weaker memory signal as indicative of a true memory than people with a more conservative response bias. The threshold that people use to determine whether a signal indicates a true memory is a relatively stable individual trait (Kantner & Lindsay, 2012, 2014), but can be shifted according to task demands and contextual effects (Aminoff et al., 2012). If these decision thresholds are contributing to the association in false memories across tasks, we would also expect to see a relationship between false alarms to new items, which is the case here. Indeed, when correcting for baseline rates of “old” responses to new lures, we found no relationships between false memories on these tasks. The implication of these findings is that people who generally expect weaker signals to reflect authentic memories could make more of a variety of false memories. But we do not see this relationship for the misinformation task, or for older adults. So the extent to which individual differences in signal threshold decisions affect general susceptibility to false memory remains to be systematically investigated. Examining whether this relationship persists when using a recall rather than recognition test could be informative in determining the contribution of internally generated errors versus individual differences in signal decision thresholds as a shared cognitive mechanism between DRM and memory conjunction tasks.

Making similar decisions regarding the strength of a memory signal across tasks does not necessarily mean that similar cognitive mechanisms contribute to the formation of false memories in the first place. As such, we also examined corrected rates of memory errors, andfound no relationship between the three tasks for younger adults. These null results are in line with previous reports showing a weak (Ball et al., 2021; Bernstein et al., 2018; Lövdén, 2003; Murphy et al., 2023; Nichols & Loftus, 2019; Platt et al., 1998; Qin et al., 2008; Zhu et al., 2013) or no relationship between these measures (Calvillo, 2014; Calvillo & Parong, 2016; Calvillo et al., 2019; Falzarano & Siedlecki, 2019; Monds et al., 2017; Ost et al., 2013; Patihis et al., 2018; Salthouse & Siedlecki, 2007; Wilkinson & Hyman, 1998). These results support the view that after accounting for response bias, false memories arising via different paradigms are driven by largely separate cognitive mechanisms.

Indeed, the false memories in our three tasks differ in various ways. False memories in the misinformation paradigm involve altering details in the original memory in response to externally provided information, DRM errors involve the inclusion of internally generated new information, and memory conjunction errors involve the miscombination of several existing details. All three errors might have distinct pathways via which they can arise. Moreover, the same type of false memory generated within the same paradigm might arise via multiple mechanisms. For instance, errors in the DRM paradigm have been accounted for theoretically by the activation-monitoring theory (Roediger et al., 2001), which suggests that during encoding, spreading activation through semantically associated memory networks results in activation of the lure, and these signals are misattributed during retrieval as indicative that the lure word was originally studied. Yet another theoretical account of DRM errors is the Fuzzy Trace Theory, which states that decay of a detail-specific verbatim memory trace and preservation of the general gist trace results in the false recognition of semantically related lures (Brainerd & Reyna, 2005). Similarly, it is thought that memory conjunction errors can occur through associative binding errors between the components of items in memory, or due to the misattribution of familiarity signals arising from the studied components comprising a conjunction word (Dodson et al., 2007).

An area for future research is to elucidate the cognitive mechanisms underpinning different memory errors, as well as the contribution of response bias, to better understand the generalizability of findings from different false memory paradigms. One avenue is to examine the extent to which experimental manipulations that increase memory errors in one paradigm are transferable to other paradigms (Thakral et al., 2019), as well as the degree to which cognitive processes reliant on constructive memory processes are related to different types of false memories (e.g., Thakral et al., 2021, 2023).

We also assessed the relationship between true memories (hit rates) across the three tasks, and found mixed results. Younger adults exhibit a relationship for both uncorrected and corrected hit rates for the memory conjunction and DRM tasks, consistent with work showing an association between memory measures using similar retrieval conditions (i.e., an old/new recognition test; see Unsworth, 2019). While the presence of this relationship in the face of no relationship for false alarm rates provides some support that unreliability is not driving the null findings, this relationship was not consistently observed for older adults in either experiment (discussed further below). Furthermore, there was no association between true memory measures for the misinformation task, and either of the other tasks. A lack of relationship between measures of true memory on the misinformation and DRM tasks is in line with prior reports using recognition tests (Bernstein et al., 2018; Calvillo & Parong, 2016; Monds et al., 2017). At best the literature reports only a small positive association between true recognition performance on these two tasks (Zhu et al., 2013). Conceptually, this lack of relationship makes sense, given the forced-choice test in the misinformation paradigm requires the retrieval of specific item information to distinguish between the two similar options. In contrast, relying on gist or familiarity signals can lead to accurate memory in the DRM and memory conjunction paradigms, when a recognition test is used. More generally, these results are reflective of recent findings that demonstrate weak and inconsistent relationships between different memory measures of the same event, which has been interpreted as demonstrating that there is no general episodic memory trait that accounts for performance across different memory tasks (Murphy et al., 2023).

### Extending findings to older adults

The main aim of the current study was to examine the relationship between false memories in various tasks in older adults. We hypothesized that either (a) age-related changes in source monitoring strategies would result in a positive association between different false memories, or (b) individual differences in age-related changes to the various cognitive mechanisms underpinning false memories would contribute to a lack of relationship between different false memories. Our findings from an online sample of older adults in Experiment 1 are in line with the second hypothesis: older adults who were more susceptible to one type of false memory were not necessarily more susceptible to other types of false memories. We replicated this result with an in-person sample in Experiment 2. This general lack of relationship between memory errors demonstrates that researchers must be wary in generalizing findings from a single false memory paradigm to all false memories when examining age differences.

Unlike younger adults, older adults did not exhibit a relationship between raw false memory rates on the DRM and memory conjunction tasks, although they still displayed a relationship between error rates for new lures on both tasks, supporting the view that response bias is a consistent individual trait (Kantner & Lindsay, 2012). Why should younger adults display a relationship between DRM and memory conjunction errors while older adults do not? Surprisingly, younger adults were more likely than older adults to falsely recognize new lures. It is possible that the online nature of Experiment 1 meant we had a sample of younger adults who were less motivated and attentive, and as such may have been less willing to shift their decision criterion across tasks (Aminoff et al., 2012). Nonetheless, it is striking that one potential mechanism that links false alarm rates across tasks – individual differences in memory decision thresholds – does not appear to persist into older age, and further work needs to be conducted to replicate these findings.

Although older adults become generally more susceptible to false memories in the majority of paradigms, the current results suggest that this increased vulnerability is not due to broad changes in a single cognitive mechanism that underpins most varieties of memory errors. Instead, it is possible that there are wide individual differences in the varying cognitive mechanisms driving false memories with age (see Devitt & Schacter, 2016). For instance, DRM errors might arise due to a reliance on memory for general rather than specific stimulus features (e.g., Koutstaal & Schacter, 1997). Memory conjunction errors could occur via reduced memory for associations between features, leading to decisions being made based on familiarity with the component features (Naveh-Benjamin, 2000; Naveh-Benjamin et al., 2003). Insufficient encoding or binding of source cues could underpin susceptibility to misinformation-based errors. It is possible that in some circumstances these mechanisms could even work against each other. For example, a loss of detailed memory with age might make one more vulnerable to gist-based errors, but resistant to misinformation errors due to reduced memory for the misleading information or an increased reliance on prior knowledge (Marche et al., 2002; Umanath & Marsh, 2012). It should be noted that in all three of the false memory paradigms examined in the current study, false memories were assessed via recognition. Different mechanisms may underpin recall and recognition, particularly in the DRM paradigm (cf. Thakral et al., 2021). The effect of aging on the relationship between different false memories elicited via recall tests remains unknown.

Continuing the trend of relationships between memory measures being weaker with age, the relationship between true memories was predominately null for older adults. Unlike younger adults, older adults showed no association between uncorrected hit rates in either experiment. In Experiment 1, a relationship was found only for corrected hit rates in the memory conjunction and DRM tasks; this relationship was not replicated in Experiment 2. As noted above, caution is advised in interpreting null relationships with the DRM task in Experiment 2, given that internal reliability was below acceptable. Nevertheless, it is possible that memory performance is less stable across different contexts with age, perhaps due to the adoption of different strategies for different memory tasks. Alternatively, the lack of relationship between the memory conjunction and DRM tasks might reflect heterogenous changes in item binding versus specific item memory with age (Naveh-Benjamin, 2000). The sum of these true and false memory relationships seems to be that episodic memory is multi-faceted, and researchers should be wary of generalizing between different memory tasks. The current results highlight the need to understand the lack of these relationships across different populations.

### Executive functioning ability

The second aim of the current study was to examine the contribution of executive functioning ability to false memories in different paradigms. In Experiment 1, we looked at working memory capacity, given prior studies have linked poorer working memory performance to increased susceptibility to false memories in the misinformation (Calvillo, 2014; Jaschinski & Wentura, 2002; Zhu et al., 2010), memory conjunction (Leding, 2012), and DRM paradigms (Peters et al., 2007; Unsworth & Brewer, 2010; Watson et al., 2005). However, in contrast to these prior findings, we found no relationship between working memory capacity and false memories in any of the three tasks, for either younger or older adults. This lack of relationship was replicated in Experiment 2, and extended to other facets of executive functioning (monitoring, inhibition, and conceptual flexibility; Latzman & Markon, 2010; Li et al., 2015). While at odds with some previous research, this null relationship between memory errors and executive functioning is in keeping with prior research showing little to no association with various measures of individual differences and susceptibility to memory errors (e.g., Calvillo et al., 2019; Patihis et al., 2018).

We also examined whether older adults with poorer executive functioning ability exhibited a positive relationship between different false memories, given executive functioning ability may contribute to age-related susceptibility to memory distortions (Butler et al., 2004; Glisky et al., 2001; Henkel et al., 1998; Plancher et al., 2009; Roediger & Geraci, 2007; Rubin et al., 1999). In both experiments, we observed a distinct relationship between different false memories depending on executive functioning capacity; however, this was in the opposite direction than expected. In Experiment 1, older adults with high executive functioning capacity exhibited a positive relationship between corrected false memories in the memory conjunction and DRM tasks, while no relationship between different false memories was seen for older adults with low executive functioning capacity. This relationship was not replicated in Experiment 2, instead a negative relationship was seen between false memories in the misinformation and memory conjunction tasks for older adults with high executive functioning ability.

It is possible that older adults with higher executive functioning capacity use compensatory processes for declines in source monitoring or memory, adopting alternative encoding and retrieval strategies that result in similar or opposing cognitive mechanisms across false memory tasks. Older adults with lower executive functioning capacity might be unable to use these compensatory mechanisms to the same degree (Bouazzaoui et al., 2014), while younger adults may not need to use compensatory strategies, accounting for the lack of relationship in false memories across tasks in those groups. However, we are hesitant to draw strong conclusions about these results, given there was no relationship between overall false memory rates and executive functioning ability, and the relationships between false memories were not replicated across experiments. Future research should validate these results, before disentangling a possible mechanism contributing to memory errors in these tasks.

### Relationship with Cognitive Failures Questionnaire

A limitation of the false memory tasks used in these experiments is that they lack ecological validity, in that they could be considered memory for ‘laboratory-based’ stimuli, which has been shown to be at least partially distinct from memory for personal, self-relevant events (e.g., Wilkinson & Hyman, 1998). In an attempt to determine whether our laboratory tests of false memories were associated with day-to-day memory errors, we ran exploratory analyses examining the relationship between false memories in each of the three tasks with the CFQ, a measure of everyday cognitive failures. No relationships were found between our false memory tasks and the CFQ, suggesting that these tasks do not readily translate to memory errors experienced in daily life. While the CFQ captures memory failures, it also covers other types of cognitive failures, including perceptual, attentional, or motor failures. As such, it is possible that the lack of relationship with the current false memory tasks may be because the CFQ represents a broader construct beyond memory errors. However, prior research has also found little to no relationship between laboratory-based memory errors and errors on tasks that better translate to the real-world (Bernstein et al., 2018; Calvillo et al., 2019; Murphy et al., 2023; Patihis et al., 2018; Qin et al., 2008). As such, the degree to which laboratory-based measures of memory errors can inform memory failures in daily life is questionable, and should continue to be examined in older adults.

One concern with null results is that it is difficult to ascertain whether there is truly no relationship, or whether the measures used are unreliable (see Bernstein et al., 2018, for discussion). In the current experiments, we could not establish internal reliability of the misinformation task, and while reliability was acceptable for the memory conjunction and DRM tasks in Experiment 1, reliability on the DRM task in Experiment 2 was slightly below acceptable. As noted above, our results with younger adults replicate a growing body of literature suggesting little to no association between various false memory measures. However, these results with older adults would benefit from additional replication attempts with measures that have a larger number of items, as well as in tasks with increased ecological validity, perhaps using tasks similar to recent attempts to examine naturalistic memory distortions in younger and older adults, (e.g., Abichou et al., 2021; Devitt et al., 2016).

In conclusion, our results replicate prior findings that false memories elicited in one task cannot be readily equated to those arising via other tasks (Bernstein et al., 2018; Calvillo, 2014; Calvillo & Parong, 2016; Calvillo et al., 2019; Monds et al., 2017; Nichols & Loftus, 2019; Ost et al., 2013; Patihis et al., 2018; Qin et al., 2008; Wilkinson & Hyman, 1998; Zhu et al., 2013). Importantly, we extend the lack of relationship between different false memories to older adults. Although overall susceptibility to false memories increases with age, there does not seem to be a common mechanism contributing to this age-related increase across false memory tasks. As such, our results concur with the view that “false memory” is a broad term that encompasses a range of types of memory distortions, and care must be taken when generalizing results across memory paradigms.

## Supplementary Information

Below is the link to the electronic supplementary material.

Supplementary file1 (DOCX 18 KB)

## Data Availability

The data and materials for all experiments are available at: https://osf.io/wfe3y/. Experiment 1 was preregistered (https://aspredicted.org/SDY_36J).

## Footnotes

1 Note that we preregistered a median split based on executive functioning scores, with subsequent correlations run for each age and executive functioning group. However, median splits have several limitations, and so we determined that a multiple regression was more appropriate. Note that when a median split analysis is run as preregistered, the overall pattern of results remains the same.
