# Supplementary material for: Age-related changes in susceptibility to false memories in different tasks
Source: Mem Cognit. 2025 Sep 2;54(3):748–66. doi: 10.3758/s13421-025-01778-x (PMC13132891; doi:10.3758/s13421-025-01778-x)
Supplement: Supplementary file 1 — Supplementary file1 (DOCX 18 KB) [file 13421_2025_1778_MOESM1_ESM.docx]

**Supplementary information**

*Executive functioning ability*

We examined whether the relationship between false memories differed according to executive functioning ability, as measured by the three DKEFS composite scores (monitoring, inhibition, and conceptual flexibility). To do so, we ran separate regressions for each pairing of false memories (using corrected proportion for DRM and memory conjunction errors), with each executive functioning score included as a predictor, and a false memory x executive functioning interaction term.

*Monitoring*

The model of misinformation errors predicting DRM errors was not significant (*R*^2^ = 0.06, *F*(3, 41) = 0.87, *p* = .463). The model with memory conjunction errors predicting DRM errors was not significant (*R*^2^ = 0.05, *F*(3, 41) = 0.71, *p* = .553). The model of misinformation errors predicting memory conjunction errors was significant (*R*^2^ = 0.21, *F*(3, 41) = 3.58, *p* = .022). Specifically, there was a significant interaction between misinformation errors and monitoring score (see Table S1). Simple effects tests reveal that for older adults with low monitoring score, there was no relationship between misinformation and memory conjunction errors. But as monitoring score increases, a negative relationship emerged between these two errors (see Table S2).

**Table S1.** Multiple regression model of predictors for errors in the memory conjunction paradigm.

| **Predictor** | ***b* [95% CI]** | ***SE*** | ***t*** | ***p*** |
| --- | --- | --- | --- | --- |
| (Intercept) | 0.12 [0.07, 0.18] | 0.03 | 4.78 | < .001 |
| Misinformation errors | -0.22 [-0.42, -0.02] | 0.10 | 2.21 | .033 |
| Monitoring score | -0.02 [-0.07, 0.04] | 0.03 | 0.66 | .516 |
| Misinformation errors x Monitoring score | -0.31 [-0.57, -0.04] | 0.13 | 2.30 | .027 |

**Table S2.** Simple effects of misinformation errors and monitoring score predicting memory conjunction errors.

| **Monitoring score** | ***b*** | ***SE*** | ***t*** | ***p*** |
| --- | --- | --- | --- | --- |
| 1 *SD* below the mean | 0.08 [-0.25, 0.42] | 0.17 | 0.49 | .626 |
| Mean | -0.22 [-0.42, -0.02] | 0.10 | 2.21 | .033 |
| 1 *SD* above the mean | -0.52 [-0.85, -0.19] | 0.16 | 3.18 | .003 |

*Inhibition*

The model of misinformation errors predicting DRM errors was not significant (*R*^2^ = 0.11, *F*(3, 41) = 1.72, *p* = .178). The model with memory conjunction errors predicting DRM errors was not significant (*R*^2^ = 0.05, *F*(3, 41) = 0.68, *p* = .570). The model of misinformation errors predicting memory conjunction errors was not significant (*R*^2^ = 0.11, *F*(3, 41) = 1.76, *p* = .17).

*Conceptual flexibility*

The model of misinformation errors predicting DRM errors was not significant (*R*^2^ = 0.003, *F*(3, 41) = 0.05, *p* = .987). The model with memory conjunction errors predicting DRM errors was not significant (*R*^2^ = 0.03, *F*(3, 41) = 0.43, *p* = .731). The model of misinformation errors predicting memory conjunction errors was significant (*R*^2^ = 0.18, *F*(3, 41) = 2.90, *p* = .047), but there was no significant effect of individual predictors (*p*s < .112).
